# Supplementary figures and images for: “I feel I have been taken seriously” Women’s experience of greater trochanteric pain syndrome treatment—A nested qualitative study
Source: PLoS One. 2022 Nov 28;17(11):e0278197. doi: 10.1371/journal.pone.0278197 (PMC9704619; doi:10.1371/journal.pone.0278197)

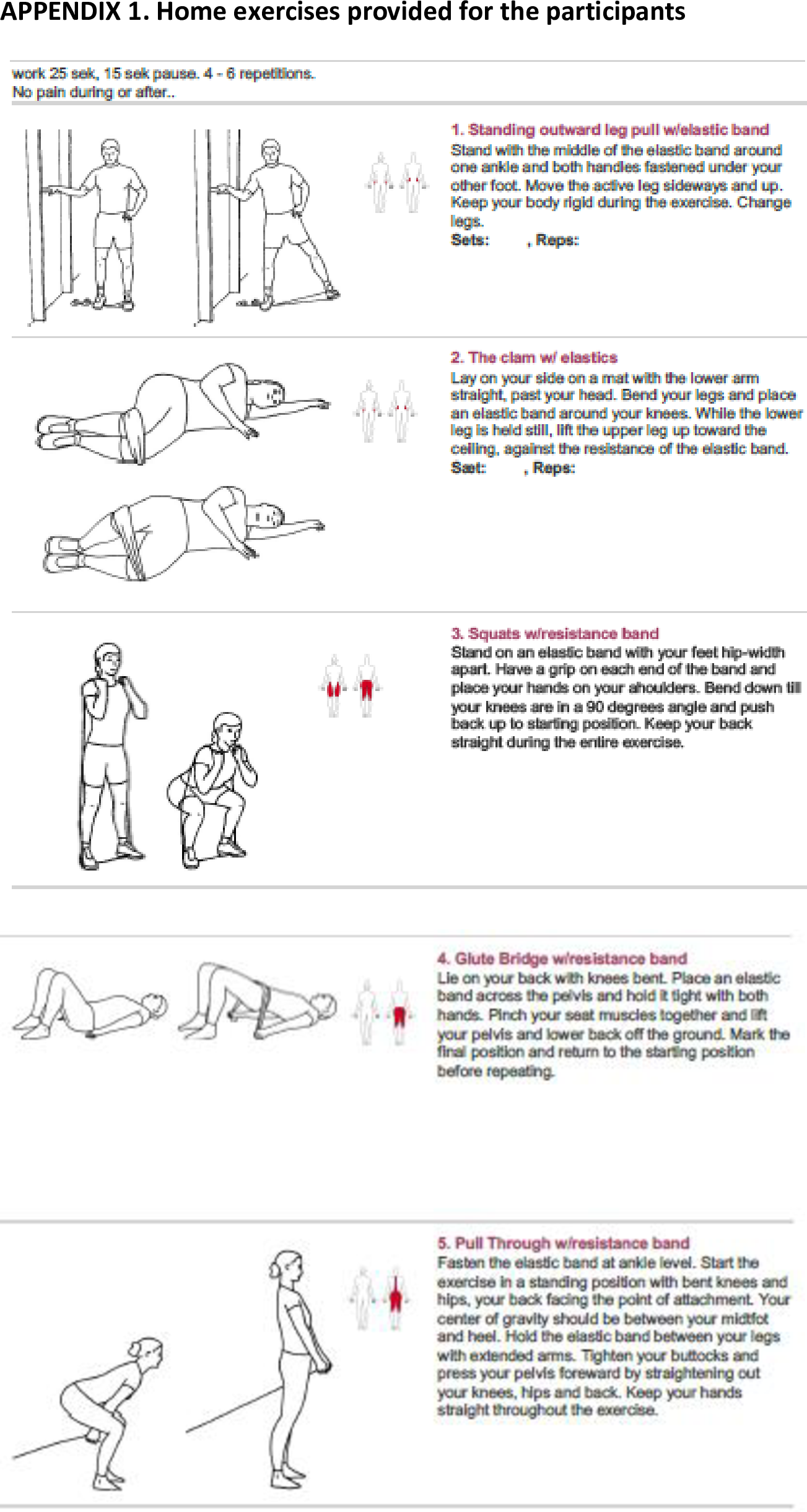

Supplement: S1 Appendix — (TIF) [file pone.0278197.s001.tif]
