# Supplementary material for: “I feel I have been taken seriously” Women’s experience of greater trochanteric pain syndrome treatment—A nested qualitative study
Source: PLoS One. 2022 Nov 28;17(11):e0278197. doi: 10.1371/journal.pone.0278197 (PMC9704619; doi:10.1371/journal.pone.0278197)
Supplement: Attachment — Submitted filename: Response to Reviewers.docx [file pone.0278197.s002.docx]

Response letter to Academic editor Rosemary Frey,

Dear Rosemary Frey,

Thank you very much for the constructive comments from Dr. Helen French and for the opportunity to revise and improve the manuscript. As recommended, we have responded to each point raised by the reviewer before uploading a 'Revised Manuscript with Track Changes' as well as a manuscript without track changes labeled 'Manuscript'. All authors have approved the final version of this revised manuscript.

We have addressed each point raised by the reviewer separately with the following structure:

1. Reviewer comment
2. Author response
3. Author actions

**Comments to the Author**

1. Is the manuscript technically sound, and do the data support the conclusions?

The manuscript must describe a technically sound piece of scientific research with data that supports the conclusions. Experiments must have been conducted rigorously, with appropriate controls, replication, and sample sizes. The conclusions must be drawn appropriately based on the data presented.

Reviewer #1: Yes

2. Has the statistical analysis been performed appropriately and rigorously?

Reviewer #1: Yes

3. Have the authors made all data underlying the findings in their manuscript fully available?

The [PLOS Data policy](https://eur01.safelinks.protection.outlook.com/?url=http%3A%2F%2Fwww.plosone.org%2Fstatic%2Fpolicies.action%23sharing&data=04%7C01%7Cjane.andreasen%40aalborg.dk%7C01e6850b0b2b492ffeb408d9a2855185%7C94fe11a337d94f50a2154fd295985b73%7C0%7C1%7C637719515833714113%7CUnknown%7CTWFpbGZsb3d8eyJWIjoiMC4wLjAwMDAiLCJQIjoiV2luMzIiLCJBTiI6Ik1haWwiLCJXVCI6Mn0%3D%7C3000&sdata=d9tbJoLFC%2FFVeiN7A2jAejCm1xMbZ%2BpmZTvENrDKqjo%3D&reserved=0) requires authors to make all data underlying the findings described in their manuscript fully available without restriction, with rare exception (please refer to the Data Availability Statement in the manuscript PDF file). The data should be provided as part of the manuscript or its supporting information, or deposited to a public repository. For example, in addition to summary statistics, the data points behind means, medians and variance measures should be available. If there are restrictions on publicly sharing data—e.g. participant privacy or use of data from a third party—those must be specified.

Reviewer #1: Yes

4. Is the manuscript presented in an intelligible fashion and written in standard English?

PLOS ONE does not copyedit accepted manuscripts, so the language in submitted articles must be clear, correct, and unambiguous. Any typographical or grammatical errors should be corrected at revision, so please note any specific errors here.

Reviewer #1: Yes

**5. Review Comments to the Author**

Reviewer #1: Thank you to the authors for this study which continues to add to the development of understanding of this condition. Some feedback is provided to improve the clarity of the paper

*Authors: Thank you very much for your time and constructive comments.*

Abstract

Pg 2, line 22-23 Suggesting adding in ‘for GTPS’ at the end of this sentence

*Authors: Agree*

***Changes to text: Education, extracorporeal shockwave therapy (ESWT) and exercise have good group level evidence for efficacy in clinical trials and are increasingly used in routine care for patients with greater trochanteric pain syndrome (GTPS).***

***____________________***

Pg 2, lines 36-37, review this wording for clarity ‘ important element of the programme to the women to be able….

*Authors: We have reviewed and changed the wording*

***Changes to text: Learning how to manage pain*** ***was experienced as the most important element of the program to the women to be able to minimize pain and manage daily life.***

_____________________
Introduction

Pg 3, line 62 add supporting references for the RCTs and qualitative studies

*Authors: We have added the supporting references.*

***Changes to text: Previous assumptions about GTPS being a mild, self-limiting condition (16,17) have therefore been disproven and the marked absence of research on interventions and experience is being addressed, as shown by a recent acceleration in the number of randomised controlled trials and qualitative studies (10,15,18–22).***

_________________

Pg 4, line 67 add detail of the sample in the qualitative study e.g. number in study, men and women

*Authors: We have added details of the sample.*

***Changes to text: A qualitative study*** ***including eleven participants (eight males and three females) found that the participants with tendinopathies were largely unaware of the mechanism of action in ESWT and that education was seen as or more important than ESWT***

***therapy (21).***

***______________***

Pg 4, line 68 review this wording for clarity ‘was seen as or more important than…’

*Authors: We have revised the wording.*

***Changes to text: A qualitative study including eleven participants (eight males and three females) found that the participants with tendinopathies were largely unaware of the mechanism of action in ESWT and*** ***that education in how to handle their condition was seen as important or even more important than ESWT therapy (21).***

***________________***

Pg 4, line 77 suggest alternative clearer wording for ‘improve their situation’

*Authors: We have rephrased the sentence for clarity.*

***Changes to text: Recent work carried out to inform trial design has begun to explore participants’ understanding of GTPS, their pain and their beliefs regarding activity and exercise (15) but we need to better understand how they experience*** ***their daily life with GTPS, how participation in a multi-modal treatment program is experienced by the women, and how it may improve their situation regarding pain and functional level.***

Methods

Pg 4, line 88 what is the specialized physiotherapist specialised in?
Also clarify in relation to the second specialist physio (JEJ)

*Authors: We have clarified in relation to both physiotherapists.*

***Changes to text:… by a physiotherapist*** ***specialized in musculo-skeletal physiotherapy (CM) at baseline, and after 12 and 26 weeks.***
***Changes to text: The ESWT, exercise therapy and additional education and advice was then performed at a physiotherapy clinic with a second physiotherapist specialized in musculo-skeletal physiotherapy (JEJ) .***

***­­­­­­­­­­­­­___________________***

Pg 5, line 95 what does a ‘thorough introduction mean and entail?

Authors:

***Changes to text: The thorough introduction consisted of an explanation to the findings and diagnose of the individual participant at inclusion by CM. During the first session by JEJ, which included the ESWT intervention, the participants were once more informed orally and in writing about the known mechanisms of how GTPS and the pain experienced by GTPS may be managed in everyday life. The education provided was to educate the participants to avoid*** ***movements and situations causing pain (10,28). The education was repeated and targeted each patient´s experience, during the following two treatment sessions. At the six-week follow up, participant experience was addressed, and any positive or negative pain experiences were used to facilitate further pain management strategies specific for the individual participant. Thereby moving from a “general” education towards a customized “personal” education specific to the individual participant´s daily context and way of life.***

_____________________

Pg 5, line 101 change ‘Those that’ to ‘Those who’

*Authors: We have changed the wording.*

***Changes to text: Those who consented were invited to interview in accordance with the purposive***

***sampling strategy, designed to ensure participants of different demographic characteristics, age,***

***severity and chronicity of GTPS were represented in order to elicit nuances and sufficient richness of data (23).***

***________________***

Pg 5, line 107, can you account for the 5 who agreed to take part, but were not interviewed? Why were they not interviewed?

*Authors: Yes, we have added this to the manuscript and moved it to the results section.*

***Changes to text: In total, 16 patients accepted the invitation to partake, and 11 were interviewed as three women had situations at home that made it inconvenient, and two women could not be reached.***

___________________

Pg 5, lines 97-98 suggesting moving the sentence on COREQ guidelines to the start of the methods section.

*Authors: We have moved the sentence to the start of the methods section.*

***Changes to text: This report was guided by the Consolidated Criteria for Reporting Qualitative Research (27). This qualitative study was nested within a cohort study (N=60) conducted in an outpatient hospital clinic and a physiotherapy clinic in Denmark.***

___________________

Pg 6, Table 1- suggest moving Table 1 participant characteristics to the results section.
In column 1 suggest changing heading to ‘location of the interview’

*Authors: We have moved Table 1 to the results section and changed the wording in column 1.*

***Changes to text:*** ***Results: In total, 16 patients accepted the invitation to partake, and 11 were interviewed. Participants were between 42 and 69 years of age, two participants had retired, one was unemployed, and the rest were employed full- or part-time (Table 1).***

***Column 1: Location for the interview.***

_________________

In row 10, is this MS Teams? Suggest being clearer e.g. ‘MS Teams teleconferencing platform’

*Authors: We have clarified.*

***Changes to text: Interview using the MS Teams teleconferencing platform.***

_________________

Pg 6, line 118 add supporting references for the literature on which you based the topic guide

*Authors: We have added references*

***Changes to text: The topic guide for the semi-structured interviews (Table 2) was based on research literature and clinical experience of the research team (10,13,14,20,31,32).***

___________________

Pg 7, line 122 – does the positive test refer to a positive COVID test?

*Authors: Yes, we have clarified.*

***Changes to text: During the COVID-19 pandemic restrictions, one interview was performed using MS Teams teleconferencing platform due to a positive COVID-19 test in the interviewee’s family.***

________________

Pg 7, Table 2: what does ‘private domain’ mean, ‘controls and follow-ups’ also not clear.

*Authors: we have altered and clarified the wording*

***Changes to text: Everyday life with GTPS; the experiences related to work life, private life and leisure time before entering the cohort study.***

***Changes to text: Experiences and reflections upon their participation in the different elements of the cohort study:***

- ***At the baseline assessment and at the follow-up assessments in the cohort study.***

_________________

Were interviews conducted in the Danish or English language. In relation to the quotations used, were these then translated into English and by whom (how can you ensure that information was not lost in translation?)

*Authors: We agree this is important to clarify. The author group consists of four researchers with mother- tongue Danish and skilled in English, two researchers with mother-tongue English/Australian and one with bilingual Danish-English tongue. We have inserted a sentence in relation the interviews performed in Danish and one in relation to the translation of the quotations- in the end of the data analysis section.*

***Changes to text: The interviews were conducted in Danish. After the analysis was conducted the used quotations were carefully translated into English by the authors to ensure the original meaning was maintained.***

__________________

Pg 10, line 177 ‘can you clarify what you mean by intimacy- is this referring to sexual intimacy. I would suggest being clearer about this as an important topic.

*Authors: Thank you, we agree and have clarified.*

***Changes to text: As the quotation illustrates, the condition was very painful and for many of the participants influenced almost every part of their daily lives, including sexual intimacy.***

__________________

Results

Pg 13, line 239, the title of this theme is not as clear as the other 3. I wonder if it is possible to elaborate the title to increase clarity?

*Authors: It is possible to change for clarity of the content. The theme “Participants´ experienced outcomes” has been changed to “The participants´ experiences of the intervention”*

***Changes to text: “The participants´ experiences of the intervention”***

__________________

Pg 13, line 254, I think ‘alfa’ should be spelt ‘alpha’

*Authors: Thank you, we have changed the spelling to ”alpha”.*

***Changes to text: “I have used them 100 %, I would say. Many of the elements, the sleeping on the back, that is alpha and omega for me, and I will never stop that.” (ID10)***

***­­­­­­­­­­­­­­­­­***
______________

Pg 14, line 264- how many are you referring to in relation to ‘a substantial number of women’

*Authors: We refer to 7 women stating this.*

***Changes to text: Seven of the women were convinced that ESWT was the significant element making the difference.***

___________________

Pg 7, Table 3. The wording of the items in the first column are not fully clear. Were these ‘themes’ devised by the researchers. Examples which I suggest require some rewording include
• ‘A plan over the treatment element and time schedule
• Plan, if it comes back
• Is a scan necessary ( is this a question?) suggest change to ‘role of imaging’?
• Follow-up on results ( does this refer to individual results or overall study results?)
• What is the difference between ‘group-based exercise training’ and ‘group-based exercise training on prescription’?
• Typing errors on ‘continuous guidance’ – again, not fully clear what this means, compared to the other headings

• Dosage, frequency and intention explicated (not sure what this word means or even exists?)
• Information or dissimilation (not sure what this word means?)
* Facebook group – context not clear

*Authors: Thank you for the suggestions and comments in relation to Table 3. The “themes” were devised of the suggestions given by the women. We have clarified the wording of the items in the first column.*

*The difference between* ‘group-based exercise training’ and ‘group-based exercise training on prescription’ is that if it is on prescription it would be free of charge for the participants over time and that it is prescribed just like medications from the physician.

***Changes to text in the first row of Table 3:***

***• An overview of the treatment and time plan
• What to do, if GTPS returns
• Role of imaging
• Follow-up on individual results

• A possibility for repeated guidance

• A written description of dosage and frequency of ESWT so that a local physiotherapist can take over the ESWT
• Information or dissemination***

- ***Facebook group for people with GTPS for sharing experiences***

_____________________
Discussion

Pg, 19, in the first line here, it is important to clarify that the experience of GTPS was in the context of a research study ( ie the cohort study), as many of the findings may relate to the effect of being in the study e.g. Hawthorne effect

*Authors: We agree, thank you for pointing this out.*

***Changes to text: This qualitative study, nested within a cohort study that assessed the outcomes of a GTPS intervention, is the first to explore how women with GTPS manage daily life and how they experience the combined treatment of education, ESWT and exercise to understand the mechanisms possibly explaining their outcomes.***

____________________

Pg 20, the therapeutic relationship also may have been influenced by the context of being in a research study and this is worth acknowledging as a limitation of the study, as it does not mimic real life

*Authors: Again, we agree on this point as well. We have added this as a limitation of the study.*

***Changes to text: A further limitation of the study was that the therapeutic relationship may have been influenced by the context of being in a research study and does therefore not mimic a real-life situation and the physiotherapists providing the treatment were specialized within musculo-skeletal conditions.***

_______________________

Pg 20, line 355 review the wording ‘the competent meeting of the individuals´ needs…’ to enhance clarity

*Authors: We have used a more precise wording.*

***Changes to text: the professional meeting of the individuals´ needs.***

________________________
Pg 21, line 362 suggesting changing ‘in the field of GTPS’ to ‘ in the management of GTPS’. In relation to the effects not being transferable to other settings, clarify if this is because is was nested in a cohort study or because of the specialist physiotherapists ( in reality, it could be both).

*Author: We agree, and we have clarified what was the message in this sentence.*

***Changes to text: Both physiotherapists in our intervention were experienced specialists within the management of GTPS, and the effects may not be directly transferable to other settings where the treating physiotherapists are not specialists within the condition.***

________________________

Pg 21, line 374, in relation to ESWT, do you mean it had a positive effect- please clarify

*Authors: We have clarified.*

***Changes to text: Most women felt that ESWT had a positive effect.***

________________________

Pg 21, line 377, add a supporting reference in relation to the qualitative study.

*Authors: We have added the reference.*

***Changes to text: These findings were not fully supported in a recent qualitative study exploring the subjective experiences of ESWT to patients with tendinopathies in general (20).***

________________________

Pg 21, line 377 is the word ‘herem’ a typing error’, please correct.

*Authors: We have reworded.*

***Changes to text: Leung and colleagues*** ***found that participants felt that self-management measures were equally or more important than ESWT to help treat their tendinopathies (20); elements which the participants in this study also found important.***

***____________________***

Pg 21, line 381, review wording ‘dialogues with advice’ for clarity

*Authors: We Have reviewed and changed for clarity.*

***Changes to text: While the pamphlet and the advice provided during the intervention were useful and appreciated by the women, the implementation of the specific exercises caused a recurrence of pain for some participants.***

­­­­­­­­­­­­­­_____________________

Pg 22, line 392, change the wording ‘relationship to’ to ‘relationship with’

*Authors: Thank you.*

***Changes to text: …..however, women in our study emphasized the value of ESWT and their relationship with the physiotherapists as well.***

***_____________________***

Pg 22, line 398 what do you mean by ‘the way of approaching women’ – does this refer to recruitment? Please clarify

*Authors: We have clarified.*

***Changes to text: The course of treatment and the way of including the women with lateral hip pain in the treatment was primarily experienced as positive and meaningful.***

_____________________

Pg 22, line 404, it is not surprising that there was alignment between the two physiotherapists’ messaging to patients it this was part of the study. Was there any exploration of the information/explanations received from other health professionals- this is a key difference between this and Stephen’s study and should be acknowledged.

Authors: Yes, some women described experiences where they had consulted a doctor, but had not received the help (added as a quotation to the results section page 10 line 171-173) they needed (page 9 line 169-170), so that is why we have the second and third key element included as recommendations for future practice. We have elaborated a little further on this as we find there may be an issue specifically present for women with GTPS as they specifically stressed that they felt taken seriously by the physiotherapists. We have added a quotation in the result section in the first theme illustrating that the women did not feel their needs were met at the physician and discussed this more in the discussion section.

***Changes to text:***

***Results section:*** ***”Just the repeated times at the doctor saying; ” Really, it keeps hurting, now I have tried this and have paused for a long time, but then it returns”. In a way you kind of feel a little like a sissy and think ”Okay, maybe it is just me”” ID4***

***Discussion section:*** ***However, participants did not feel that their physicians had met their needs. This issue does not seem to be specific for women with GTPS as recent qualitative studies exploring other musculo-skeletal conditions also describe similar experiences in their target populations (38–40). But the women in this study also stressed that they felt they had been taken seriously by the physiotherapists which may imply that they had not previously felt taken seriously. However, we did not explicitly ask if their physician had taken them seriously and future research should look into this.***

_____________________

Pg 22, line 416, in relation to saturation, please clarify what you mean. How would you have determined data saturation? Suggest reviewing and referring to recent papers in relation to the concepts/controversies around data saturation.

Authors: We have looked further into the literature and especially the recent controversies regarding saturation in qualitative research that has been discussed, both regarding the many different ways of defining it and whether it is a legitimate argument or an artificial and constructed argument for stopping the data collection (Saunders 2018, Thorne 2020). Braun and Clarke (2013) state that the term saturation invokes a more positivist model of qualitative research and the researcher should consider which approach should be taken (Brown and Clarke 2013). Recent papers by Saunders et al. (2018) and Thorne (2020) discuss different approaches to saturation and especially Thorne problematizes the term and states that it is more important to assess whether there is sufficient depth, richness, detail and coherence within the reported findings to make interpretations and conclusions relevant and credible in relation to complex clinical phenomena. We find that our data and analysis have provided depth, richness and coherence within the findings and that the interpretations and conclusions are relevant and credible in relation to complex clinical field of GTPS. Therefore, we have looked more into data richness and depth, and coherence in the findings.

**Changes to text:** ***Braun and Clarke (2013) state that the term saturation invokes a more positivist model of qualitative research and the researcher should consider which approach should be taken (Brown and Clarke 2013). Recent papers by Saunders et al. (2018) and Thorne (2020) discuss different approaches to saturation (Saunders, Thorne). Especially Thorne problematizes the term and states that it is more important to assess whether there is sufficient depth, richness and coherence within the reported findings to make interpretations and conclusions relevant and credible in relation to complex clinical phenomena. We find that our data and analysis have provided depth, richness and coherence within the findings and that the interpretations and conclusions are relevant and credible in relation to complex clinical field of GTPS.***

______________________

Pg 23, Please present study limitations more clearly in this section. The heading methodological considerations is not clear. Perhaps changes to study strengths and limitation?

*Authors: We have changed the heading and presented the strengths and limitations more clearly.*

***Changes to text:***

***Study strengths and limitations.***

***The participants met the predefined criteria of variation in age, symptoms and duration. The analytical process was carried out by two authors in the initial phase, first independently and thereafter in collaboration. Subsequently the author group was involved to ensure analytical sensitivity and agreement. This process was performed and presuppositions were reflected upon to achieve trustworthy and credible findings*** (34)***. This rigorous analytical process was considered a strength of the study. Further, the authors included both non-clinical and clinical researchers, thereby possessing knowledge within the clinical as well as the theoretical and methodological field. The 11 interviews provided rich data and views, however, as this was a rather small sample, saturation cannot be guaranteed, neither can it be guaranteed that all viewpoints were expressed. Braun and Clarke (2013) state that the term saturation invokes a more positivist model of qualitative research and the researcher should consider which approach should be taken*** (34)***. Recent papers by Saunders et al. (2018) and Thorne (2020) discuss different approaches to saturation*** (43,44)***. Especially Thorne problematizes the term and states that it is more important to assess whether there is sufficient depth, richness and coherence within the reported findings to make interpretations and conclusions relevant and credible in relation to complex clinical phenomena. We find that our data and analysis have provided depth, richness and coherence within the findings and that the interpretations and conclusions are relevant and credible in relation to complex clinical field of GTPS.*** ***It may be considered a limitation that no interviewees were presented with the results of this qualitative study although this could have validated the findings. A further limitation of the study was that the therapeutic relationship may have been influenced by the context of being in a research study and does therefore not mimic a real-life situation and the physiotherapists providing the treatment were specialized within musculo-skeletal conditions. Therefore, transferring the findings to other contexts should be done cautiously*** (34)***.***

_______________________

Pg 23, line 417 do you mean the results of this qualitative study or the results of the cohort study, please clarify

*Authors: We have clarified.*

***Changes to text: No interviewees were presented with the results of this qualitative study, although this could have validated the findings.***

The conclusion should explicitly state that the experience of women in this study is nested within a cohort study

*Authors: We agree, and we have added a sentence in the start of the conclusion.*

***Changes to text***: ***This qualitative study, nested within a cohort study assessing the outcomes of a treatment intervention, is the first to explore how women with GTPS manage daily life and how they experience the combined treatment of education, ESWT and exercise to understand the mechanisms possibly explaining their outcomes.***

______________________

Pg 23, review wording ‘are important knowledge…’ to enhance clarity

*Authors: We have reworded for clarity.*

***Changes to text: These new findings regarding how women with GTPS experienced and managed pain in daily life, and how they experience and adapt to treatment elements, are important improvements to inform clinical practice for women with GTPS.***

6. PLOS authors have the option to publish the peer review history of their article ([what does this mean?](https://eur01.safelinks.protection.outlook.com/?url=https%3A%2F%2Fjournals.plos.org%2Fplosone%2Fs%2Feditorial-and-peer-review-process%23loc-peer-review-history&data=04%7C01%7Cjane.andreasen%40aalborg.dk%7C01e6850b0b2b492ffeb408d9a2855185%7C94fe11a337d94f50a2154fd295985b73%7C0%7C1%7C637719515833724069%7CUnknown%7CTWFpbGZsb3d8eyJWIjoiMC4wLjAwMDAiLCJQIjoiV2luMzIiLCJBTiI6Ik1haWwiLCJXVCI6Mn0%3D%7C3000&sdata=rwTjT1SGTYgpDpsmFFgrVgaaul9lT53ZmniGJBlzrMc%3D&reserved=0)). If published, this will include your full peer review and any attached files.


If you choose “no”, your identity will remain anonymous but your review may still be made public.


**Do you want your identity to be public for this peer review?** For information about this choice, including consent withdrawal, please see our [Privacy Policy](https://eur01.safelinks.protection.outlook.com/?url=https%3A%2F%2Fwww.plos.org%2Fprivacy-policy&data=04%7C01%7Cjane.andreasen%40aalborg.dk%7C01e6850b0b2b492ffeb408d9a2855185%7C94fe11a337d94f50a2154fd295985b73%7C0%7C1%7C637719515833724069%7CUnknown%7CTWFpbGZsb3d8eyJWIjoiMC4wLjAwMDAiLCJQIjoiV2luMzIiLCJBTiI6Ik1haWwiLCJXVCI6Mn0%3D%7C3000&sdata=LE13qT9Qop0VXXlT3NLCAj0XXR7xAyIQDL3cd4F9HOs%3D&reserved=0).

Reviewer #1: **Yes:** Helen French

[NOTE: If reviewer comments were submitted as an attachment file, they will be attached to this email and accessible via the submission site. Please log into your account, locate the manuscript record, and check for the action link "View Attachments". If this link does not appear, there are no attachment files.]

While revising your submission, please upload your figure files to the Preflight Analysis and Conversion Engine (PACE) digital diagnostic tool, [https://pacev2.apexcovantage.com/](https://eur01.safelinks.protection.outlook.com/?url=https%3A%2F%2Fpacev2.apexcovantage.com%2F&data=04%7C01%7Cjane.andreasen%40aalborg.dk%7C01e6850b0b2b492ffeb408d9a2855185%7C94fe11a337d94f50a2154fd295985b73%7C0%7C1%7C637719515833734028%7CUnknown%7CTWFpbGZsb3d8eyJWIjoiMC4wLjAwMDAiLCJQIjoiV2luMzIiLCJBTiI6Ik1haWwiLCJXVCI6Mn0%3D%7C3000&sdata=F28DhnkIwng0YFZrVblLm21ugZi9RJUXbFjp5d8HU3Y%3D&reserved=0). PACE helps ensure that figures meet PLOS requirements. To use PACE, you must first register as a user. Registration is free. Then, login and navigate to the UPLOAD tab, where you will find detailed instructions on how to use the tool. If you encounter any issues or have any questions when using PACE, please email PLOS at [figures@plos.org](mailto:figures@plos.org). Please note that Supporting Information files do not need this step.
