# Supplementary material for: “I feel I have been taken seriously” Women’s experience of greater trochanteric pain syndrome treatment—A nested qualitative study
Source: PLoS One. 2022 Nov 28;17(11):e0278197. doi: 10.1371/journal.pone.0278197 (PMC9704619; doi:10.1371/journal.pone.0278197)
Supplement: Attachment — Submitted filename: Response to reviewers_14062022 .docx [file pone.0278197.s003.docx]

Response to reviewers and editor

Thank you very much for the constructive comments from editor, reviewer 1 and reviewer 2, and for the opportunity to revise and improve the manuscript. As recommended, we have responded to each point raised by the reviewers before uploading a 'Revised Manuscript with Track Changes' as well as a manuscript without track changes labeled 'Manuscript'.

We have addressed each point raised by the reviewers separately with the following structure:

1. Reviewer comment
2. Author response and actions

**Comments to the Author**

1. If the authors have adequately addressed your comments raised in a previous round of review and you feel that this manuscript is now acceptable for publication, you may indicate that here to bypass the “Comments to the Author” section, enter your conflict of interest statement in the “Confidential to Editor” section, and submit your "Accept" recommendation.

Reviewer #1: All comments have been addressed

Reviewer #2: (No Response)

2. Is the manuscript technically sound, and do the data support the conclusions?

The manuscript must describe a technically sound piece of scientific research with data that supports the conclusions. Experiments must have been conducted rigorously, with appropriate controls, replication, and sample sizes. The conclusions must be drawn appropriately based on the data presented.

Reviewer #1: Yes

Reviewer #2: Partly

3. Has the statistical analysis been performed appropriately and rigorously?

Reviewer #1: Yes

Reviewer #2: Yes

4. Have the authors made all data underlying the findings in their manuscript fully available?

The [PLOS Data policy](https://eur03.safelinks.protection.outlook.com/?url=http%3A%2F%2Fwww.plosone.org%2Fstatic%2Fpolicies.action%23sharing&data=05%7C01%7C%7C5a53f2ba2896455e926308da2bf30aad%7C5968b90c51a64f088b4750ffffbe2e4f%7C0%7C0%7C637870620697251150%7CUnknown%7CTWFpbGZsb3d8eyJWIjoiMC4wLjAwMDAiLCJQIjoiV2luMzIiLCJBTiI6Ik1haWwiLCJXVCI6Mn0%3D%7C2000%7C%7C%7C&sdata=voWhoTkE0ZOPpCIq4zjmDtM5hoxRrGOycvU2AG40Hgk%3D&reserved=0) requires authors to make all data underlying the findings described in their manuscript fully available without restriction, with rare exception (please refer to the Data Availability Statement in the manuscript PDF file). The data should be provided as part of the manuscript or its supporting information, or deposited to a public repository. For example, in addition to summary statistics, the data points behind means, medians and variance measures should be available. If there are restrictions on publicly sharing data—e.g. participant privacy or use of data from a third party—those must be specified.

Reviewer #1: No

Reviewer #2: No

5. Is the manuscript presented in an intelligible fashion and written in standard English?

PLOS ONE does not copyedit accepted manuscripts, so the language in submitted articles must be clear, correct, and unambiguous. Any typographical or grammatical errors should be corrected at revision, so please note any specific errors here.

Reviewer #1: Yes

Reviewer #2: Yes

6. Review Comments to the Author

Please use the space provided to explain your answers to the questions above. You may also include additional comments for the author, including concerns about dual publication, research ethics, or publication ethics. (Please upload your review as an attachment if it exceeds 20,000 characters)

**Reviewer #1**: Dear authors, many thanks for submitting this revision and addressing previous feedback. I have some minor suggestions only, some of which are more related to wording/language and grammar, based on some of the additional text added to the revision.

*Author response and actions: Thank you very much for your response and your suggestions, we have addressed each of your comments below*.

Pg 5, lines 98-100, this sentence which has been added by the authors needs a review in relation to clarity
The thorough introduction consisted of an explanation to the findings and diagnose of the individual participant at inclusion by CM
Suggest (if I understand correctly that this is what you mean?)
'This introduction consisted of an explanation regarding the clinical findings and diagnosis of each participant at inclusion by CM'

*Author response and actions: Thank you, we have revised as suggested*

The inserted paragraph could be more succinctly written as suggested here
During the first session by JEJ, which included the ESWT intervention, participants were once more informed, including the provision of written information, about the known mechanisms of how GTPS and associated pain may be managed in everyday life. Participants were educated on how to avoid movements and situations causing pain (10, 28). The education was repeated and individualised for each patient´s experience, during the following two treatment sessions. At the six-week follow up, participant experience was addressed, and any positive or negative pain experiences were used to facilitate further pain management strategies specific for the individual participant. Thereby moving from a “general” education towards a customized “personal” education specific to the individual participant´s daily context and way of life.

*Author response and actions: We agree and have revised as suggested.*

Table 1: final column heading should read ‘pain relief’ rather than ‘pain relieve’. Were those descriptors based on some form of global rating of change scale? If so, please add a legend to explain it and provide a supporting reference for the scale.
In column 4, job situation, suggest the word unemployed (if that’s what ‘not in job’ means), as used in the text (line 153). Align the legend (line 157) under the table.

*Author response and actions: Thank you, we have changed to “pain relief” and “unemployed”.* *We did not use a specific global rating scale, we asked the women about it and put them into one of these four categories according to their answers.*

*We have therefore inserted and aligned the legend under the table with the following:*

**^#^ The women were asked how they experienced their improvement, and their response was categorized into one of four categories; no improvement, minor to moderate improvement, considerable improvement or full or nearly full recovery.**


Pg 16, I wonder if the term ‘reduce activity levels’ is better here than ‘slow down’ (just a suggestion)

*Author response and actions: We agree and have revised*

Pg 20, lines 347-349. I think sentence either needs to be two sentences or separated by a semi-colon
'Pain education and management was a central element of the intervention for the women as a measure of success was their ability to minimize pain and manage their daily life, during and after treatment'.

*Author response and actions: We agree and have inserted a semi-colon.*

Pg 21, lines 378-381. I don’t think changing the word here to ‘professional’ is appropriate here, unless participants specifically mentioned the professional behaviour of the physiotherapist (which I did not see in any illustrative quotes).

I suggest the following
‘The therapeutic alliance, characterized by acknowledgement of the individuals’ situation, addressing individuals’ needs, and a secure therapeutic relationship, may be the mechanisms that supported the development of intrinsic motivation and self-efficacy as described by Ryan and Deci (42)’.

*Author response and actions: Thank you for your suggestion, we have changed accordingly.*


Pg 22, line 385-387. Suggest rewording that sentence to the following
'Both physiotherapists in our intervention were experienced specialists in managing GTPS, and the effects may not be directly transferable to other settings where the treating physiotherapists are not specialists in the management of this condition.'

*Author response and actions: Thank you, we have reworded as suggested.*

Pg 24, line 446, Suggest removing the word ‘Especially’ before ‘Thorne’

*Author response and actions: We agree and have removed* ‘Especially’.

Pg 25, line 476, Suggest the wording ‘additions to the current evidence’ is preferred than ‘improvements’

*Author response and actions: We fully agree and have reworded the sentence for clarity.*

Check all quotes in the text and Table 3 to ensure consistency in formatting e.g. quotation marks, ID number in brackets.

*Author response and actions: We have scrutinized to ensure consistency in Table 3 and have put ID numbers in brackets, thank you.*

**Reviewer #2:** Abstract:
Background: change to: “A combination of education, ESWT and exercise are…”. Line 24-26 should not be in the Background section of the abstract. Rather, use this space to explain why the aim is to look at the experiences of ‘their daily life’ (in addition to treatment).

*Author response and actions: We have discussed this in the author group, and we find this is an important information in advance. However, we also find it important to explain why the aim is to look at the experiences of their daily life as well and we have therefore added this (The abstract is still less than 300 words).*

Methods: I would suggest including important qualitative aspects like: were the interviews in-person? Were they video/audio recorded.

*Author response and actions: We have added these aspects.*

Rather than including ‘a six-step approach’, I would suggest spelling out the 6 steps, or change to something like: “transcripts were coded and analysed during a inductive thematic approach.”

*Author response and actions: We have reworded as suggested.*

Findings: include numbers of themes. (1)xx, (2) xxx, (3) xxx.

*Author response and actions: We have included numbers of themes as suggested.*

Line 36-40 mostly repeats the themes identified in line 33-36. Further, the names of the themes should/could be self-explanatory. I would suggest rewriting this section.

*Author response and actions: We agree and have removed most of the text after numbering and naming the themes.*

Conclusion: Lin 41-43 suggest that the aim was to explore (1) the experience and management of hip pain, and (2) experience and adapting to treatment. Firstly, I still do not quite understand why authors are exploring: daily living & experiences of treatment. Do authors mean that the daily living is explored as a result of the treatment? Or in general? If it is in general, I think authors should make a clearer rational to why these questions are combined in 1 research question. Secondly, please ensure your background, objectives, results, conclusions align with the specific research aim. Line 43-45: The reviewer would suggest to remove this section, as this is not a conclusion that is closely aligned with your findings.

*Author response and actions: We hope our introduction in the abstract does clarify our reasoning – we did take a holistic approach and wanted to include contextual experiences of daily life and how the women managed their condition as the starting point for understanding their experiences with the treatment program. We have chosen to keep the last sentence in the conclusion however we have reworded to align this more with our findings.*

Introduction

Aim: Line 81-83- objective; do authors mean that they explore experiences, feelings or perceptions of the impact of hip pain due to GTPS on daily life?

*Author response and actions: We hope that our previous explanations clarify that we reasoned that we would achieve more insightful and contextualised knowledge from the women by the approach chosen.*

Authors should be clear in what it is they are assessing. Authors should be clearer in the aim that this study looked at women with GTPS that were included in a cohort study/ after receiving treatment as part of a larger study, and that this qual study mostly reflects on this cohort study.

*Author response and actions:*

*We agree that we could clarify this further. In the introduction we write the following that we “*need to better understand how they experience their daily life with GTPS, how participation in a multi-modal treatment program is experienced by the women, and how it may improve their situation regarding pain and functional level.”

*We have clarified this by adding it to the aim as well:* The aim of this study was to explore how women with greater trochanteric pain syndrome, **participating in a multi-modal treatment program**, experience and manage both their daily life and the combined treatment of education, ESWT and exercise.

Methods:
Line 120: could you include more information about the background of the qualitative researcher?

*Author response and actions: We have revised the Methods section and added more information. We have added information about the qualitative researcher to the manuscript.*

Line 121: was the topic guide developed a priori? What did it include (questions, prompts, both)?

*Author response and actions: The topic guide was developed a priori. The interview guide included questions and prompts were used if necessary, to support or guide the participants. This is added to the manuscript.*

Did recruitment, data collection and analysis proceed concurrently?

*Author response and actions: Recruitment and data collection was finalized before the analysis was initiated. This is also added to the manuscript.*

How did authors ensure data saturation?

*Author response and actions: As described in the methods section we strived to ensure rich data descriptions but did not strive for data saturation. We further discuss this in the discussion section. We sincerely hope that our explanations and references clarify this approach, an approach that is accepted within thematic analysis as it is questioned whether it is possible to be sure and as a researcher guarantee that you have covered all aspects of a topic. We have made no further changes to the manuscript after we revised the methodological considerations in the discussion section after the first revision as recommended by reviewer 1.*

Results:
The results section is very long and wordy (page 10-16). The reviewer suggests that authors rewrite the results section by integrating quotes in the theme explanations, to minimise repetition of findings.

*Author response and actions: We have scrutinized the results section and revised and condensed to some degree, however we find that the validation of the findings is a very important part of the results section as we find that this gives a deeper insight in to the lives of the female participants in the study. Further reviewer 1 has not commented further on the results section after the first revision was submitted to PLOS ONE. We sincerely hope that this approach is accepted.*

Conclusion:
Line 460-461: this study explored experiences, as such, it did not “understand the mechanisms possibly explaining their outcomes”.

*Author response and actions: Thank you for this comment. We do explore the experiences and perspectives of the women, but we did also use this knowledge to try to understand mechanisms possibly explaining their outcomes. We do state “possibly” as we do not provide any evidence for causal explanations in this nested qualitative study. However, we have revised and removed the last part of the sentence.*

Authors should be cautious in inferring that findings can be used as a framework for an individualized patient education, treatment and evaluation strategy. What is this based on?

*Author response and actions: Thank you for this response. We agree that we should be a little more cautious than to suggest this knowledge as sufficient evidence to suggest it used as a framework. We have therefore revised the last sentence in the conclusion.*

The conclusion should make clear that findings are in the context of the cohort study, and the specific treatment that the participants received.

*Author response and actions: We fully agree and have cautiously done this throughout the manuscript. We have made it clear in the abstract, in the aim, in the methods- and discussion section and finally it is stated in the start of the conclusion.*

7. PLOS authors have the option to publish the peer review history of their article ([what does this mean?](https://eur03.safelinks.protection.outlook.com/?url=https%3A%2F%2Fjournals.plos.org%2Fplosone%2Fs%2Feditorial-and-peer-review-process%23loc-peer-review-history&data=05%7C01%7C%7C5a53f2ba2896455e926308da2bf30aad%7C5968b90c51a64f088b4750ffffbe2e4f%7C0%7C0%7C637870620697251150%7CUnknown%7CTWFpbGZsb3d8eyJWIjoiMC4wLjAwMDAiLCJQIjoiV2luMzIiLCJBTiI6Ik1haWwiLCJXVCI6Mn0%3D%7C2000%7C%7C%7C&sdata=PmFR70N%2Bpj89a54FVvIEphpR1A1ZS%2F1StpASJWT98H8%3D&reserved=0)). If published, this will include your full peer review and any attached files.


If you choose “no”, your identity will remain anonymous but your review may still be made public.


**Do you want your identity to be public for this peer review?** For information about this choice, including consent withdrawal, please see our [Privacy Policy](https://eur03.safelinks.protection.outlook.com/?url=https%3A%2F%2Fwww.plos.org%2Fprivacy-policy&data=05%7C01%7C%7C5a53f2ba2896455e926308da2bf30aad%7C5968b90c51a64f088b4750ffffbe2e4f%7C0%7C0%7C637870620697251150%7CUnknown%7CTWFpbGZsb3d8eyJWIjoiMC4wLjAwMDAiLCJQIjoiV2luMzIiLCJBTiI6Ik1haWwiLCJXVCI6Mn0%3D%7C2000%7C%7C%7C&sdata=tfqK%2Be9yJ5hkvSiTzgorLq2K%2B2Dpi7O30HprJ1XG2NY%3D&reserved=0).

Reviewer #1: **Yes:** Helen P French

Reviewer #2: No

[NOTE: If reviewer comments were submitted as an attachment file, they will be attached to this email and accessible via the submission site. Please log into your account, locate the manuscript record, and check for the action link "View Attachments". If this link does not appear, there are no attachment files.]

While revising your submission, please upload your figure files to the Preflight Analysis and Conversion Engine (PACE) digital diagnostic tool, [https://pacev2.apexcovantage.com/](https://eur03.safelinks.protection.outlook.com/?url=https%3A%2F%2Fpacev2.apexcovantage.com%2F&data=05%7C01%7C%7C5a53f2ba2896455e926308da2bf30aad%7C5968b90c51a64f088b4750ffffbe2e4f%7C0%7C0%7C637870620697251150%7CUnknown%7CTWFpbGZsb3d8eyJWIjoiMC4wLjAwMDAiLCJQIjoiV2luMzIiLCJBTiI6Ik1haWwiLCJXVCI6Mn0%3D%7C2000%7C%7C%7C&sdata=npi%2BnwsQPoqd4%2FXDFviuOc50OI5xgLUd7yvoSorNVYk%3D&reserved=0). PACE helps ensure that figures meet PLOS requirements. To use PACE, you must first register as a user. Registration is free. Then, login and navigate to the UPLOAD tab, where you will find detailed instructions on how to use the tool. If you encounter any issues or have any questions when using PACE, please email PLOS at [figures@plos.org](mailto:figures@plos.org). Please note that Supporting Information files do not need this step.

*In compliance with data protection regulations, you may request that we remove your personal registration details at any time.* [*(Remove my information/details)*](https://eur03.safelinks.protection.outlook.com/?url=https%3A%2F%2Fwww.editorialmanager.com%2Fpone%2Flogin.asp%3Fa%3Dr&data=05%7C01%7C%7C5a53f2ba2896455e926308da2bf30aad%7C5968b90c51a64f088b4750ffffbe2e4f%7C0%7C0%7C637870620697251150%7CUnknown%7CTWFpbGZsb3d8eyJWIjoiMC4wLjAwMDAiLCJQIjoiV2luMzIiLCJBTiI6Ik1haWwiLCJXVCI6Mn0%3D%7C2000%7C%7C%7C&sdata=6hQQTiPCt0DJ8u%2F8Y1j3YGSwygGfKtlXqXgtofmjgYQ%3D&reserved=0)*. Please contact the publication office if you have any questions.*
