# Supplementary material for: “I feel I have been taken seriously” Women’s experience of greater trochanteric pain syndrome treatment—A nested qualitative study
Source: PLoS One. 2022 Nov 28;17(11):e0278197. doi: 10.1371/journal.pone.0278197 (PMC9704619; doi:10.1371/journal.pone.0278197)
Supplement: Attachment — Submitted filename: Response to reviewers_24.09.2022.docx [file pone.0278197.s004.docx]

September 2022

Response letter,

Thank you very much for the constructive comments from the reviewers and for the opportunity to revise and improve the manuscript. As recommended, we have responded to each point raised by the reviewers before uploading a 'Revised Manuscript with Track Changes' as well as a manuscript without track changes labeled 'Manuscript'.

We have addressed each point raised by the reviewers separately with the following structure:

1. Reviewer comment
2. Author response and actions

6. Review Comments to the Author

Please use the space provided to explain your answers to the questions above. You may also include additional comments for the author, including concerns about dual publication, research ethics, or publication ethics. (Please upload your review as an attachment if it exceeds 20,000 characters)

Reviewer #1: I have no further comments- well done to the authors who have addressed previous comments sufficiently.

*Response: Thank you very much.*

Reviewer #3: Comments to author
Thank you for this study which adds knowledge about the experiences of women with GTPS.

*Response: Thank you very much.*

Abstract
Background: Line 27: in the sentence “Exploring women’s experience … should help understand … “ suggest changing the word should to may

*Response: We have revised to the suggested change*

Methods: Line 34: in the sentence “Transcripts were coded and analyse during an …” suggest changing the word during to using

*Response: We have revised as suggested*

Introduction
Line 50 spell out GTPS in full when at beginning of sentence

*Response: Revised*

Line 60: change the wording of “therefore been disproven” to softer response along the lines of “this may not be the case” recent evidence seems to support this is not the case etc.

*Response: We have revised as suggested to a softer response*

Lines 70-74: I can see why you are introducing therapeutic alliance but feel this section needs a little more justification to include here and why it is of interest in your study.

*Response: We have added further justification for the focus on therapeutic alliance.*

*Changes to text: This is important to successfully addressing GTPS, given that all successful interventions have included elements of education and exercise that require active participation, learning and behavior change. Therefore, aspects of therapeutic alliance as a contributor to the experiences of effect and confidence in managing GTPS are important to explore further.*

Methods
Line 88: is the protocol for the cohort study published? If so, could reference here.

*Response: We agree, but the protocol has not been published. The findings of the cohort study are being processed for publication at the moment.*

Line 42: you have chosen social constructive approach. Perhaps more information needed her as to why this was the most appropriate choice rather than others used in qualitative health studies such as qualitative content approach or phenomenological approach.

*Response: We have added information on why we find this approach beneficial.*

*Changes to text:*

*The theoretical approach was social constructivism to explore insights regarding how people interact with their social world, and focused on the participants’ experiences in their particular contexts (33,35).* *This focus can therefore help us to explore how the women themselves construct their knowledge, understanding and management of the GTPS condition in their everyday life.*

Lines 170-173: The 5 themes listed. I suggest you consider re-naming themes 3 and 5 as the way they currently are is just a summary of the findings rather than alluding to the direction of the theme. Suggest re-naming 3 and 5 so they better reflect the findings contained within these themes. Eg. theme 3, information helps/reassures/motivates is the general feeling I am getting; theme 5, women want more is the general message I am reading?

*Response: We have re-named theme 3 and 5 as suggested by the reviewer as we have pointed more to the direction of the theme.*

*Theme 3:* ***The participants´ experiences of the intervention*** ***– information is key***

*Theme 5:* ***The women´s perspectives on improving and expanding the intervention***

Discussion
Line 408: How? Provide some suggestions for the reader for how exercise may be addressed more carefully or some relevant strategies/directions they could take away

*Response: We have added suggestions*

*Changes to text:*

*The issues related to non-adherence and low compliance is well-known in general (43) and in physiotherapy as well regarding non-supervised exercises (44,45) and should be addressed carefully, i.e by a thorough dialogue with the women regarding the type and intensity of pain being acceptable during exercise as well as carefully selecting which and how many exercises to provide to each woman.*

Limitations
The sample has experienced symptoms of varying duration ( 6 months – 10 years) and varying pain (3-8 rating) – I would assume this also has impacts upon the findings and may deserve a mention.

*Response: Thank you for this comment, we agree, this deserves a mention. We have added this.*

*Changes to text:*

*Finally, it should be noticed that the women have experienced symptoms of varying duration and varying pain.*

Also, a comment in the discussion about whether your theoretical approach was useful or had any limitations may be beneficial?

*Response: We agree and have added this to the section:*

*Changes to text:*

*We find the theoretical approach in this study useful as the analysis of the data using this approach helped us to explore and emphasize that the women individually constructed their understanding and management of the GTPS condition in their given contexts.*

On behalf of the author group,

Jane Andreasen
