# Supplementary material for: “I feel I have been taken seriously” Women’s experience of greater trochanteric pain syndrome treatment—A nested qualitative study
Source: PLoS One. 2022 Nov 28;17(11):e0278197. doi: 10.1371/journal.pone.0278197 (PMC9704619; doi:10.1371/journal.pone.0278197)
Supplement: Attachment — Submitted filename: Response letter_01112022.docx [file pone.0278197.s005.docx]

Dear editor Rosemary Frey and reviewers,

Thank you very much for letting me revise again. I want to apologize for the not fully revised manuscript that I forwarded to you.

6. Review Comments to the Author

Please use the space provided to explain your answers to the questions above. You may also include additional comments for the author, including concerns about dual publication, research ethics, or publication ethics. (Please upload your review as an attachment if it exceeds 20,000 characters)

Reviewer #1: (No Response)

Reviewer #3: Thank you for addressing comments.
Please update the new theme titles for consistency throughout the paper - in abstract results section and in paper lines 175-178 where the themes are listed.

Response: I apologize once again for the inconvenience and I have revised as suggested, both in the abstract and in the manuscript.

Kind regards,

Jane Andreasen
